# Association between reduced left ventricular ejection fraction and peritoneal dialysis related peritonitis: a single center retrospective cohort study in Japan

**DOI:** 10.1038/s41598-023-49744-4

**Published:** 2023-12-20

**Authors:** Makoto Yamaguchi, Takaaki Obayashi, Naoto Kobayashi, Naoki Izumi, Masaaki Nagai, Hironobu Nobata, Akimasa Asai, Keisuke Kamiya, Hirokazu Sugiyama, Hiroshi Kinashi, Shogo Banno, Masahiko Ando, Takahiro Imaizumi, Yoko Kubo, Takayuki Katsuno, Takuji Ishimoto, Yasuhiko Ito

**Affiliations:** 1 Department of Nephrology and Rheumatology, Aichi Medical University, Nagakute, Japan; 2 Department of Nephrology, Narita Memorial Hospital, Toyohashi, Japan; 3 Data Coordinating Center, Department of Advanced Medicine, Nagoya University Hospital, Nagoya, Japan; 4 Department of Preventive Medicine, Nagoya University Graduate School of Medicine, Nagoya, Japan; 5 Department of Nephrology and Rheumatology, Aichi Medical University Medical Center, Okazaki, Aichi Japan

**Keywords:** Kidney, Chronic kidney disease, End-stage renal disease

## Abstract

We present a single-center retrospective analysis of 228 Japanese patients with peritoneal dialysis, in which we examined whether reduced left ventricular ejection fraction (LVEF) is a risk factor for peritonitis development. Time-dependent multivariable-adjusted Cox proportional hazards models revealed that reduced LVEF (LVEF < 50% vs. preserved LVEF ≥ 50%, hazard ratio (HR) 2.10; 95% confidence interval (CI) 1.16–3.82) was associated with peritonitis. Qualitatively, similar associations with reduced LVEF (< 50%) were observed for enteric peritonitis (adjusted HR 7.68; 95% CI 2.51–23.5) but not for non-enteric peritonitis (adjusted HR 1.15; 95% CI 0.54–2.44). Reduced LVEF is associated with a significantly higher risk of subsequent peritonitis, particularly enteric peritonitis. These results indicate that patients with reduced LVEF may be at risk of enteric peritonitis from bowel sources caused by intestinal involvement due to cardiac dysfunction.

## Introduction

Peritonitis is a serious complication of peritoneal dialysis (PD) that is associated with significant morbidity, catheter loss, transfer to hemodialysis, transient loss of ultrafiltration, permanent membrane damage, and occasionally death^1–4^. Various strategies have been suggested to reduce the risk of peritonitis. However, PD-related peritonitis rates have not adequately improved^2–4^.

Hypokalemia, constipation, and usage of anti-gastric acid agents (H2 receptor antagonists [H2RA] or proton pump inhibitor [PPI]) are some of the previously reported risk factors for PD-related peritonitis^5^, which can cause impairment of intestinal movement, alterations in the intestinal microbiota, and bacterial translocation, leading to the development of peritonitis^6–10^. Cardiovascular dysfunction, a major complication in patients undergoing dialysis, is reportedly common in such patients^11,12^ and is associated with a higher incidence of mortality and hospitalization^13–19^. Left ventricular (LV) dysfunction can directly lead to cardiac failure and is strongly associated with poor survival in patients undergoing dialysis with constant hypervolemia^20,21^.

Hypervolemia due to heart failure impacts the gastrointestinal system by inducing hemodynamic changes affecting the gut morphology, function, and permeability^22–24^. However, no previous studies have assessed the relationship between heart failure and PD-related peritonitis. Therefore, in the present study, we aimed to assess whether patients with PD and reduced left ventricular ejection fraction (LVEF) are vulnerable to developing PD-related peritonitis.

The results of the present study may provide useful clinical information for identifying patients with PD who are at high risk of developing peritonitis by examining their LV function.

## Methods

**Figure 1:**
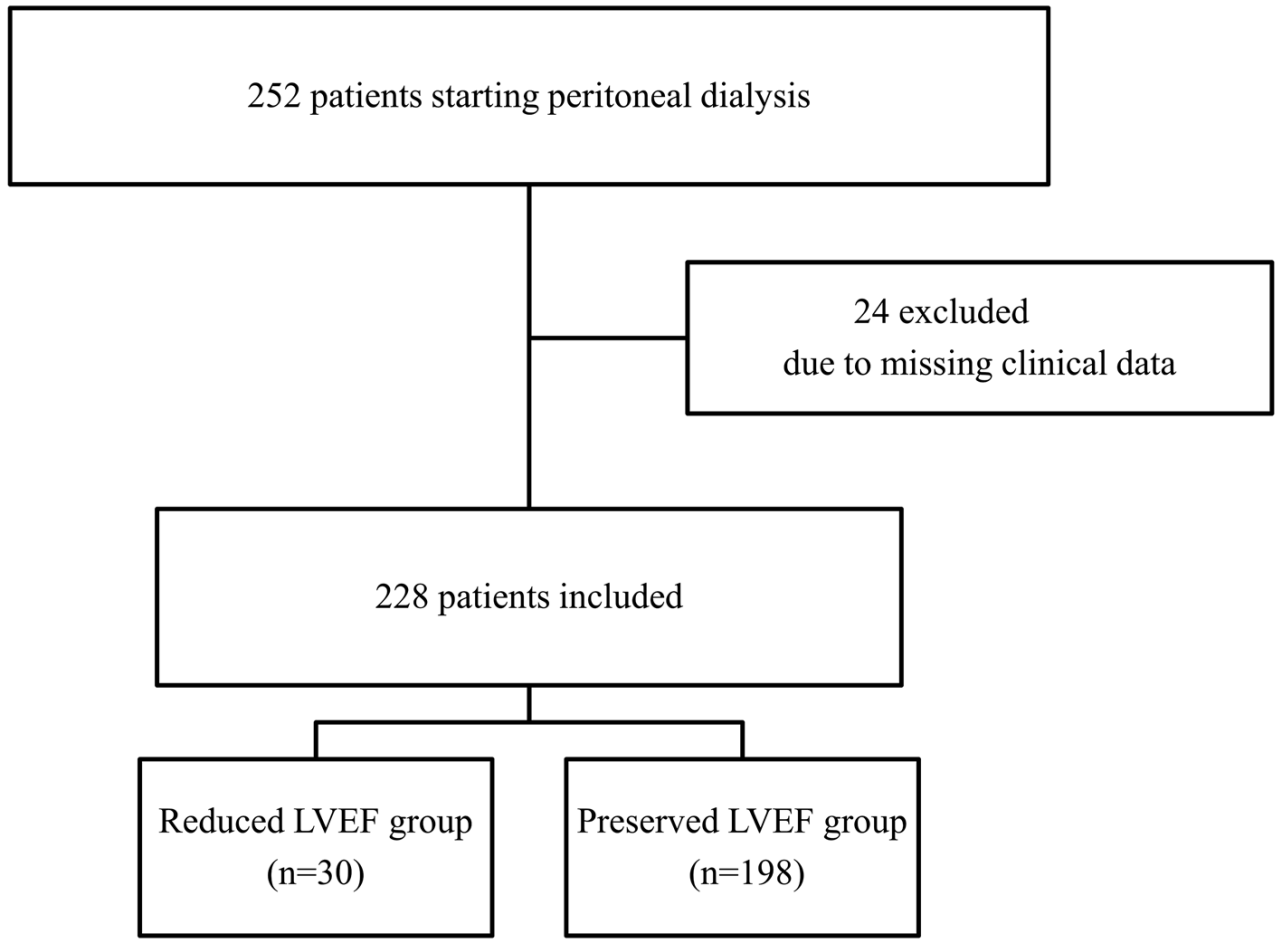
Flow diagram showing patient selection.

This study included patients aged ≥ 20 years who began PD as renal replacement therapy between January 1997 and December 2017 at Narita Memorial Hospital. Ultrasound echocardiography (UCG) was routinely performed within one month of PD initiation. Among 252 patients, 24 (9.5%) were excluded because of missing UCG data and clinically relevant information, and 228 patients with PD (90.5%) were finally included in the analysis (Fig. 1).

### Ethics

The study protocol was approved by the Ethics Committee of the Narita Memorial Hospital (Approval Number 29-12-01). The study was conducted in accordance with Declaration of Helsinki and the Ethical Guidelines for Medical and Health Research Involving Human Subjects enacted by the Ministry of Health, Labour and Welfare of Japan [https://www.mhlw.go.jp/content/001077424.pdf]. Due to the retrospective nature of the study, the need for patients’ informed consent was waived by the Ethics Committee of the Narita Memorial Hospital.

### Measurements

The design of the present study has been described in detail in a previous report^25^. Briefly, baseline characteristics at the start of PD included age, sex, body mass index (BMI), previous atherothrombotic events (coronary heart disease, heart failure, stroke, aortic aneurism and/or peripheral vascular disease requiring intervention or hospital admission), comorbidities (hypertension and diabetes mellitus), cause of kidney disease (diabetic nephropathy, glomerulonephritis, and renal sclerosis), laboratory data (hemoglobin, serum albumin, serum potassium, C-reactive protein, brain natriuretic peptide (BNP) level, and estimated glomerular filtration rate [eGFR], estimated using the equation recently generated by the Japanese Society of Nephrology: eGFR [mL/min/1.73 m^2^] = 194 × Scr^−1.094^ × Age^−0.287^ × 0.739 [if female]^26^), urine volume (mL/day), peritoneal transport characteristics (dialysate/plasma ratio of creatinine at 240 min during peritoneal equilibration test), daily peritoneal ultrafiltration rate (calculated as the difference between the volume of total dialysate infused and volume drained over 24 h), domestic pets, smoking, constipation (defined as a state of using laxatives), and usage of PPI or H2RA, as previously reported^27^, cardiothoracic ratio on chest X-ray, and findings of ultrasonic echocardiography. Furthermore, the follow-up data on BMI and urine volume (mL/day) were collected every 12 months.

PD-related peritonitis was diagnosed when at least two of the following conditions were met: (1) abdominal pain and/or cloudy dialysis effluent, which are clinical features of peritonitis; (2) dialysis effluent white cell count > 100/µL or > 0.1 × 10^9^/L (after a dwell time of at least 2 h), with > 50% polymorphonuclear leukocytes; (3) positive dialysis effluent culture^5^.

The anonymized data set is shown in Table S1.

### Echocardiography

Ultrasonic echocardiography was performed according to the American Society of Echocardiography recommendations. LVEF was measured using the modified Sympson method^28^. We stratified the patients into two LVEF groups, i.e., reduced LVEF group (LVEF < 50%), and preserved LVEF group (LVEF ≥ 50%), as reported previously^29,30^. LV mass was calculated using the formula recommended by the American Society of Echocardiography^28^, and indexed based on the body surface area. The diameters of the inferior vena cava (IVC) were measured at approximately 3 cm before merging with the right atrium at end expiration (IVC max) and at inspiration with sniffing (IVC min)^31^. The collapsibility of the IVC (IVCC) was calculated as IVC_max_ minus IVC_min_ divided by IVC_max_.

### Exposure and outcomes

The primary exposure of interest was LVEF at baseline and the first episode of peritonitis from any cause was the primary outcome of interest. Patients were followed up until the first episode of peritonitis, or censoring events such as loss to follow-up, death (cardiovascular disease, malignancy, infection, and others), PD withdrawal, or end of the follow-up for this study, whichever happened earlier.

Furthermore, we classified peritonitis into “enteric” and “non-enteric” peritonitis corresponding to previous reports^32,33^. Specifically, we defined enteric peritonitis as being caused by enteric organisms such as enteric bacilli (*Escherichia*, *Klebsiella*, *Serratia*, *Proteus*, etc.) and enterococcus (*Enterococcus faecalis*, *Enterococcus faecium*, etc.)^34^. We defined other peritonitis cases as non-enteric peritonitis. Incident enteric and non-enteric peritonitis were defined as secondary outcomes.

Additionally, outcomes including PD withdrawal and its causes (PD-related peritonitis, inadequate solute clearance, impairment of activities of daily living, fluid overload, and kidney transplantation) were obtained.

### Statistics

Differences in clinical characteristics and outcomes according to the LVEF groups (reduced LVEF (< 50%) and preserved LVEF (≥ 50%)) were compared using the Wilcoxon rank-sum test or Fisher’s exact test.

To identify predictors independently associated with the outcome, we examined potential confounding factors that have previously been reported as clinically important risks for PD-related peritonitis occurrence^5^ by using unadjusted and time-dependent multivariable-adjusted Cox proportional hazard (CPH) regression models. The models were adjusted for the following potential confounders: baseline data, including age (years), sex, diabetes mellitus, constipation, serum albumin (g/dL), serum potassium (mEq/L), use of PPI, daily ultrafiltration rate (mL), reduced LVEF (< 50%); and follow-up data, including BMI and urine volume (mL/day) at every 12 months.

Furthermore, we employed a stratified analysis to account for each potential confounder, including age, sex, constipation, use of PPI, diabetes mellitus, serum albumin, serum potassium, BMI, urine output, and daily ultrafiltration rate, with reduced LVEF as the exposure of interest. We constructed a forest plot to demonstrate the hazard ratio (HR) for the development of enteric peritonitis in each stratum.

To elucidate the dose-dependent association between LVEF and incidence of peritonitis, restricted cubic spline functions with three knots placed at the 10th, 50th, and 90th percentiles of LVEF were used. Furthermore, we conducted a similar analysis after classifying patients into the enteric and non-enteric peritonitis groups.

The proportional hazard assumption for covariates was tested using scaled Schoenfeld residuals. The cumulative probability for the occurrence of the first episode of peritonitis from any cause, enteric and non-enteric, was calculated using the Kaplan–Meier method and log-rank test.

Continuous variables are expressed as the medians and interquartile ranges, while categorical variables are expressed as numbers and proportions. Significance was set at *P* < 0.05. Statistical analyses were conducted using the Stata software (version 15.0; StataCorp LP, College Station, TX, USA) and JMP software version 14.0.0 (SAS Institute, Cary, NC, USA).

## Results

### Study participants and clinical characteristics

This study included 228 PD patients, including 30 (13.2%) patients in the reduced LVEF group (LVEF < 50%) and 198 (86.8%) patients in the preserved LVEF group (LVEF ≥ 50%). The baseline characteristics of the two groups are summarized in Table 1. The reduced LVEF group had a higher proportion of patients with a previous history of coronary heart disease and heart failure, higher BNP, cardiothoracic ratio on chest X-ray, LV mass index, LV end-diastolic dimension, LV end-systolic dimension, and IVC (max and min) on echocardiography than the preserved LVEF group. Conversely, the IVCC in patients with reduced LVEF was lower than that in patients with preserved LVEF. Other baseline factors did not differ significantly between the two groups.

**Table 1:** Comparison of baseline characteristics between the reduced LVEF (n = 30) and preserved LVEF (n = 198) groups.

	Reduced LVEF (LVEF < 50%) (n = 30)	Preserved LVEF (LVEF ≥ 50%) (n = 198)
Age (year)	62 (49–72)	64 (56–72)
Male (N (%))	25 (83.3)	138 (69.7)
Body mass index (kg/m^2^)	22.7 (20.6–23.5)	22.2 (19.8–24.7)
Previous atherothrombotic event	15 (50.0)	48 (24.2)
Coronary heart disease*	7 (23.3)	13 (6.6)
Heart failure*	15 (50.0)	18 (9.1)
Stroke	6 (20.0)	22 (11.1)
Aortic aneurism and/or peripheral vascular disease	2 (6.7)	3 (1.5)
Comorbidities		
Hypertension	27 (90.0)	160 (80.8)
Diabetes mellitus	18 (60.0)	93 (47.0)
Cause of kidney disease		
Diabetic nephropathy	17 (56.7)	103 (52.0)
Glomerulonephritis	7 (23.3)	56 (28.3)
Renal sclerosis	1 (3.3)	14 (7.1)
Others	5 (16.7)	25 (12.6)
Laboratory data		
Hemoglobin (g/dL)	10.1 (9.4–11.1)	9.9 (9.0–11.0)
Serum albumin (g/L)	3.4 (2.8–3.9)	3.4 (3.0–3.8)
Serum potassium (mEq/L)	4.3 (3.6–4.9)	4.2 (3.6–4.7)
eGFR (mL/m/1.73 m^2^)	6.9 (5.7–9.3)	7.2 (5.7–8.9)
CRP (mg/dL)	0.3 (0.1–2.6)	0.2 (0.1–0.7)
BNP (pg/mL)*	457 (182–1403)	193 (121–322)
Urine volume (mL/day)	1200 (807–1750)	1000 (700–1315)
D/P creatinine	0.70 (0.65–0.84)	0.67 (0.58–0.77)
Daily ultrafiltration rate (mL)	580 (400–730)	500 (290–715)
Domestic pet	4 (13.3)	44 (22.2)
Smokers (current/ex-)	13 (43.3)	62 (31.3)
Constipation (use of laxative)	21 (70.0)	144 (72.7)
Medications		
Anti-hypertensive drugs	25 (83.3)	158 (79.8)
PPI	10 (33.3)	62 (31.3)
H2RA	4 (13.3)	31 (15.7)
Chest X-ray		
Cardiothoracic ratio (%)*	54 (49–56)	49 (45–53)
Echocardiography		
LVEF (%)*	42 (38–47)	66 (58–73)
LVMI (g/m^2^)*	127 (106–165)	109 (88–130)
LVDd (mm)*	53 (50–55)	39 (33–43)
LVDs (mm)*	35 (32–39)	24 (21–28)
IVC_max_ (mm)*	18 (16–21)	13 (12–15)
IVC_min_ (mm)*	11 (9–13)	6 (5–7)
IVCC (%)*	42 (30–51)	54 (47–62)

Median (interquartile range) and categorical values are expressed as numbers (proportions).

Conversion factors for units: SCr in mg/dL to μmol/L, × 88.4; eGFR (mL/min/1.73 m^2^) = 194 × Scr^-1.094^ × Age^-0.287^ × 0.739 (if female),

*HF* Heart failure, *eGFR* estimated glomerular filtration rate, *PPI* Proton pomp inhibitor, *H2RA* H2-receptor antagonist, *CRP* C-reactive protein, *BNP* Brain natriuretic peptide, *D/P* Dialysate/plasma ratio, *LVEF* Left ventricular ejection fraction, *LVMI* Left ventricular mass index, *LVDd* Left ventricular end-diastolic dimension, *LVDs* Left ventricular end-systolic dimension, *IVC* Inferior vena cava, *IVC*_*max*_ Maximal diameter of inferior vena cava at expiration, *IVC*_*min*_ Minimal diameter of inferior vena cava with sniffing, *IVCC* Inferior vena cava collapsibility.

*P < 0.05.

### Outcome data

#### Peritonitis from any cause (primary outcome)

**Figure 2:**
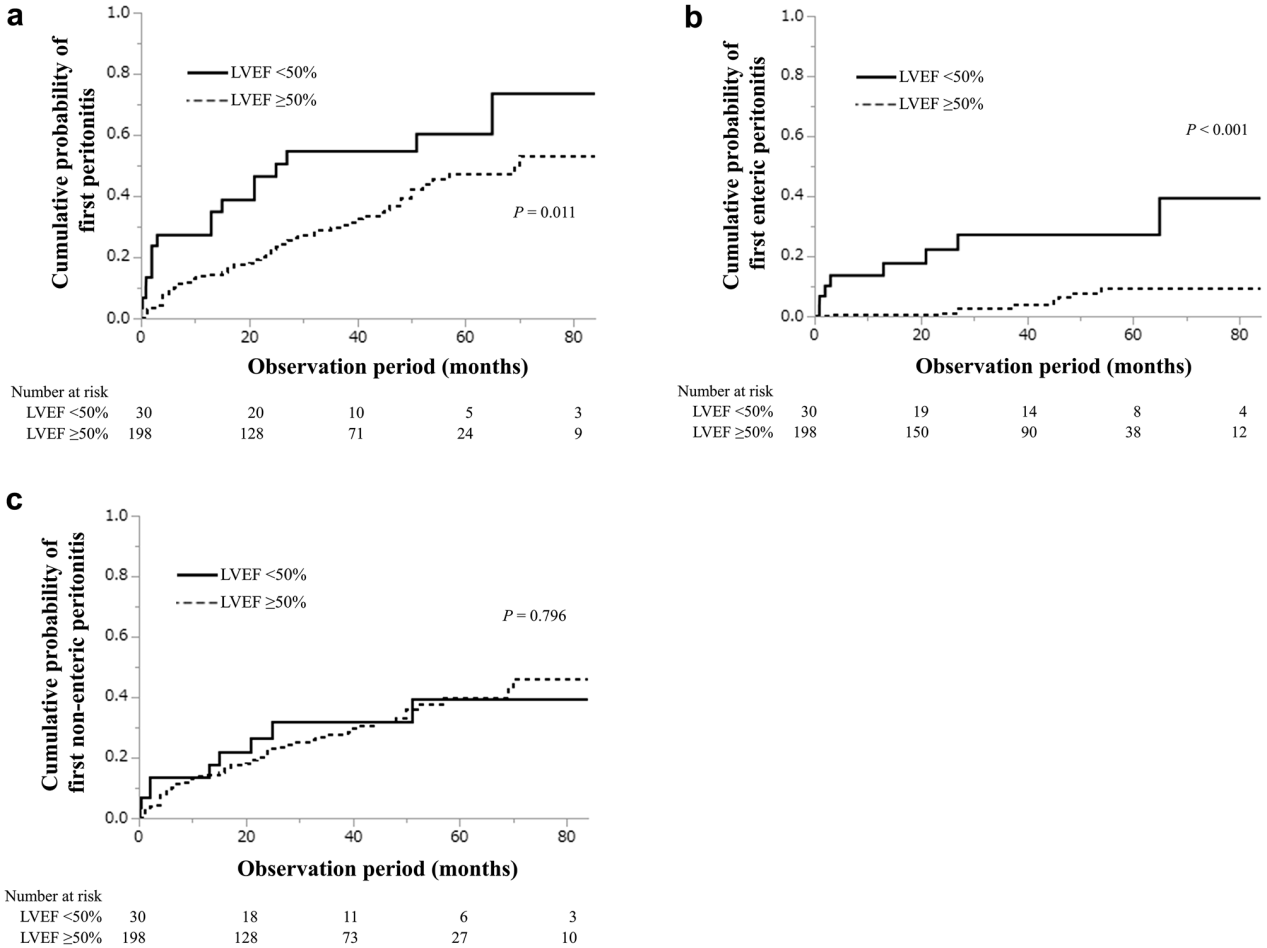
Cumulative probability of all-cause peritonitis (**a**), enteric peritonitis (**b**), and non-enteric peritonitis (**c**) between the two LVEF groups. LVEF, left ventricular ejection fraction.

**Figure 3:**
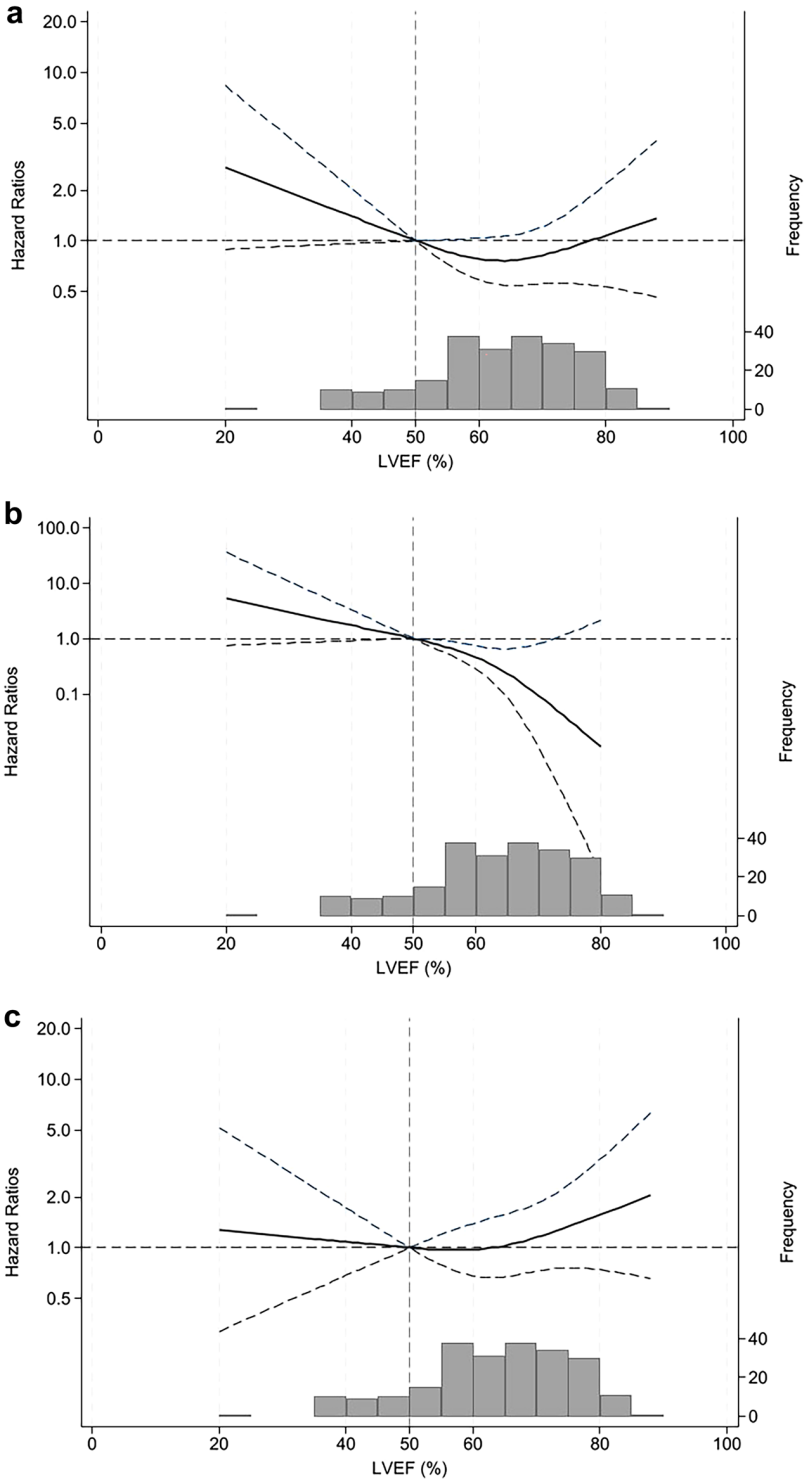
Restricted cubic spline curve for the association of all-cause peritonitis (**a**), enteric peritonitis (**b**), and non- enteric peritonitis (**c**), adjusted for age (years), sex, diabetes mellitus, constipation, serum albumin (g/dL), serum potassium (mEq/L), use of PPI, and reduced LVEF (< 50%) as covariate. PPI, proton pump inhibitor; LVEF, left ventricular ejection fraction.

During the follow-up period (median, 36 months; interquartile range, 19–57 months), 17 (56.7%) and 67 (33.8%) patients in the reduced and preserved LVEF groups, respectively, developed peritonitis at least once (Table 2). The incidence of peritonitis was 0.25 and 0.12 person-year in the reduced LVEF and preserved LVEF groups, respectively. The cumulative probabilities of the first episode of peritonitis at 1, 3, and 5 years were 0.27, 0.45, and 0.60, respectively, in the reduced LVEF group, and 0.15, 0.30, and 0.48, respectively, in the preserved LVEF group; this indicated that the reduced LVEF group had a higher risk of developing peritonitis than the preserved LVEF group (log-rank test: *P* = 0.011; Fig. 2a). In the unadjusted models, diabetes mellitus, lower serum albumin levels, PPI use, and reduced LVEF (LVEF < 50%) were significantly associated with the first episode of peritonitis (Table 3). Time-dependent multivariable-adjusted CPH models further showed that PPI use (HR 1.85; 95% confidence interval [CI] 1.19–2.89), and reduced LVEF (vs. preserved LVEF; HR 2.10; 95% CI 1.16–3.82) were associated with peritonitis (Table 3). A multivariable-adjusted restricted cubic spline model confirmed the nonlinear association between LVEF and incidence of peritonitis (Fig. 3a), suggesting that reduced LVEF was associated with a higher risk of peritonitis.

**Table 2:** Comparison of characteristics of peritonitis between the reduced LVEF (n = 30) and preserved LVEF (n = 198) groups.

	Reduced LVEF (LVEF < 50%) (n = 30)	Preserved LVEF (LVEF ≥ 50%) (n = 198)
Peritonitis incidence (any cause)		
Peritonitis (at least one episode)*	17 (56.7)	67 (33.8)
Peritonitis (≥ 2 episode)*	11 (36.7)	31 (15.7)
Observation period (months)	35 (13–59)	36 (20–56)
Classification of peritonitis (enteric and non-enteric)	n = 17	n = 67
Enteric peritonitis*	8 (47.1)	9 (13.4)
*Escherichia coli*	1	1
*Klebsiella* species	1	1
*Serratia marcescens*	1	0
*Proteus mirabilis*	1	1
*Enterococcus* species	2	0
Others	2	6
Non-enteric peritonitis	9 (30.0)	58 (29.3)
*Staphylococcus aureus*	5	7
Coagulase-negative *Staphylococcus* species	0	8
*Streptococcus* species	1	5
Others	1	13
Culture-negative	3	25
Concomitant exit-site infection or tunnel infection	1 (5.9)	2 (3.0)

Median (interquartile range) and categorical values are expressed as numbers (proportions).

*LVEF* Left ventricular ejection fraction.

*P < 0.05.

**Table 3:** Predictors of peritonitis from any cause.

	Peritonitis (n = 84)	
	Unadjusted HR (95% CI)	Adjusted HR (95% CI)
Baseline data		
Age (per 1 year)	1.01 (0.99–1.03)	1.01 (0.99–1.03)
Male (vs. female)	1.37 (0.84–2.23)	1.2 (0.71–2.08)
Diabetes (vs. non-diabetes)	1.86 (1.20–2.88)*	1.42 (0.89–2.28)
Serum potassium (per 1.0 mEq/L)	0.98 (0.74–1.31)	1.03 (0.76–1.39)
Serum albumin (per 1.0 g/dL)	0.51 (0.35–0.75)*	0.64 (0.41–1.00)
Constipation (vs. non-constipation)	1.15 (0.71–1.87)	1.07 (0.65–1.77)
PPI use (vs. non-PPI)	1.96 (1.28–3.01)*	1.85 (1.19–2.89)*
Daily ultrafiltration rate (per 1.0 mL)	1.00 (1.00–1.00)	1.00 (1.00–1.00)
Reduced LVEF (vs. preserved LVEF)	1.97 (1.15–3.35)*	2.10 (1.16–3.82)*
Follow-up data		
BMI (per 1.0 kg/m^2^)	1.00 (0.95–1.06)	1.02 (0.96–1.08)
Urine volume (per 1 mL/day)	0.83 (0.62–1.12)	0.86 (0.60–1.25)

Data are presented as HR, 95% CI, and *P* value from Cox proportional hazard regression analyses. Adjusted for baseline data (age, sex, diabetes mellitus, serum potassium, serum albumin, constipation, use of PPI, daily ultrafiltration, and reduced LVEF) and follow-up data (BMI and urine volume).

*HR* Hazard ratio, *CI* Confidence interval, *PPI* Proton pump inhibitor, *LVEF* Left ventricular ejection fraction, *BMI* Body mass index.

*P < 0.05.

#### Enteric and non-enteric peritonitis (secondary outcomes)

During the follow-up period, 17 (56.7%) and 67 (33.8%) patients developed incident enteric and non-enteric peritonitis, respectively. Among the 17 patients with enteric peritonitis, the proportion of patients with enteric peritonitis in the reduced LVEF group was higher than that of the preserved LVEF group (Table 2).

The pattern of association between LVEF groups and the incidence of enteric peritonitis was qualitatively similar to that with peritonitis from any cause; that is, compared with patients in the preserved LVEF group, those in the reduced LVEF group had a higher cumulative probability of developing enteric peritonitis (log-rank test: *P* < 0.001; Fig. 2b). Furthermore, unadjusted and time-dependent multivariable-adjusted CPH models demonstrated that the reduced LVEF group was significantly associated with the occurrence of enteric peritonitis (adjusted HR 7.68; 95% CI 2.51–23.5, Table 4). A nonlinear association between LVEF and enteric peritonitis was also verified in a multivariable-adjusted restricted cubic spline model (Fig. 3b), suggesting that reduced LVEF was associated with a higher risk of enteric peritonitis.

**Table 4:** Predictors of enteric and non-enteric peritonitis.

	Enteric peritonitis (n = 17)		Non-enteric peritonitis (n = 67)	
	Unadjusted HR (95% CI)	Adjusted HR (95% CI)	Unadjusted HR (95% CI)	Adjusted HR (95% CI)
Baseline data				
Age (per 1 year)	1.00 (0.96–1.04)	1.02 (0.98–1.06)	1.02 (0.99–1.04)	1.00 (0.98–1.03)
Male (vs. female)	7.93 (1.05–60.1)*	7.87 (0.95–64.9)	1.01 (0.60–1.69)	0.93 (0.53–1.64)
Diabetes (vs. non-diabetes)	0.98 (0.38–2.54)	0.66 (0.21–2.05)	2.06 (1.25–3.38)*	1.65 (0.97–2.81)
Serum potassium (per 1.0 mEq/L)	1.60 (0.57–1.98)	0.63 (0.29–1.34)	0.97 (0.71–1.33)	1.13 (0.82–1.57)
Serum albumin (per 1.0 g/dL)	1.12 (0.45–2.82)	1.48 (0.50–4.45)	0.44 (0.29–0.68)	0.54 (0.33–0.87)*
Constipation (vs. non-constipation)	0.71 (0.26–1.91)	0.46 (0.16–1.34)	1.31 (0.75–2.30)	1.36 (0.76–2.43)
PPI use (vs. non-PPI)	1.65 (0.64–4.29)	2.13 (0.75–6.02)	2.06 (1.27–3.32)*	1.81 (1.10–2.97)*
Daily ultrafiltration rate (per 1.0 mL)	1.00 (1.00–1.00)	1.00 (1.00–1.00)	1.00 (1.00–1.00)	1.00 (1.00–1.00)
Reduced LVEF (vs. preserved LVEF)	6.08 (2.34–15.8)*	7.68 (2.51–23.5)*	1.10 (0.54–2.21)	1.15 (0.54–2.44)
Follow-up data				
BMI (per 1.0 kg/m^2^)	1.02 (0.91–1.14)	1.04 (0.90–1.21)	1.00 (0.94–1.06)	1.01 (0.94–1.07)
Urine volume (per 1 mL/day)	1.83 (0.73–4.57)	1.23 (0.47–3.21)	0.74 (0.54–1.01)	0.89 (0.60–1.32)

*HR* Hazard ratio, *CI* Confidence interval, *PPI* Proton pump inhibitor, *LVEF* Left ventricular ejection fraction, *BMI* Body mass index.

Data are presented as HR, 95% CI, and *P* value from Cox proportional hazard regression analyses. Adjusted for baseline data (age, sex, diabetes mellitus, serum potassium, serum albumin, constipation, use of PPI, daily ultrafiltration, and reduced LVEF) and follow-up data (BMI and urine volume).

*P < 0.05.

In contrast, no significant association was observed between LVEF and non-enteric peritonitis. No significant difference was observed in the cumulative incidence of non-enteric peritonitis between the two groups with respect to LVEF (log-rank test: *P* = 0.796, Fig. 2c). Unadjusted and time-dependent multivariable-adjusted CPH models showed no significant association between LVEF groups and non-enteric peritonitis (adjusted HR 1.15; 95% CI 0.54–2.44, Table 4). Additionally, no significant association was found between LVEF and non-enteric peritonitis in the multivariable-adjusted restricted cubic spline model (Fig. 3c).

Furthermore, a forest plot demonstrating the HR for the development of enteric peritonitis in each potential confounder indicated similar associations throughout, except for age, sex, and daily ultrafiltration rate (Supplementary Fig. S1).

#### PD withdrawal

A total of 22 (73.3%) and 145 (73.3%) patients in the reduced and preserved LVEF groups, respectively, withdrew from PD. The reasons for PD withdrawal, such as mortality events of all causes, indicating that the cause of death, were not significantly different between the LVEF groups (Table S2).

## Discussion

This study showed that reduced LVEF was significantly associated with the development of PD-related peritonitis. In particular, a significant association was observed with the development of enteric peritonitis and not with non-enteric peritonitis. These results suggest that patients with reduced LVEF may be at risk of developing enteric peritonitis caused by intestinal conditions triggered by cardiac dysfunction, providing clinically useful information for physicians to cautiously monitor peritonitis caused by enteric microorganisms in patients with cardiac dysfunction. To our knowledge, this is the first study to evaluate the association between cardiac function and PD-related peritonitis.

Among the previously reported modifiable risk factors for PD peritonitis^6,35^, gastrointestinal conditions, such as constipation^7^, and hypokalemia^6,8–10^, have been reported to be associated with peritonitis due to enteric organisms. Furthermore, emerging data suggests that gastric acid suppression, particularly with H2RA, is a modifiable risk factor for enteric peritonitis in patients undergoing PD, although the risk of peritonitis associated with PPI is sporadically reported^25,36^. Several mechanisms have been speculated to foster peritonitis in PD, including induction of gastrointestinal dysmotility^37^ and intestinal bacterial overgrowth^38^. Consequently, the translocation of bacteria from the intestine to the peritoneal cavity may cause peritonitis. This mechanism is similar to that of spontaneous bacterial peritonitis in cases of liver cirrhosis^39^.

Therefore, it is important to detect gastrointestinal conditions that may increase vulnerability for development of peritonitis; however, this has not been entirely evaluated.

One retrospective single-center study, which included 580 patients with PD, showed an association between overhydration, as measured by bioimpedance, and a higher incidence of peritonitis and infections from enteric organisms^40^. Although the results were comparable with those of the present study, the previous study did not evaluate the relationship between echocardiographic cardiac dysfunction and peritonitis.

Previous reports have shown that patients with heart failure experience alterations in the morphology, function, and bacterial flora of the intestine^19^ through the following pathophysiological mechanisms: increased venous pressure imposes relative ischemia on the intestinal microvilli leaving enterocytes at the villus tip susceptible to ischemic injury. Moreover, ischemic conditions in the intestine may cause a reduced barrier function and the translocation of potentially pathogenic microorganisms, and visceral congestion and the generation of relatively ischemic conditions may cause environmental alterations in the bacterial microbiome of the intestinal lumen^41–43^. Intestinal overgrowth of pathogenic bacteria and increased intestinal permeability may also occur. Given the pathogenic gut flora and increased intestinal permeability, we consider that cardiac dysfunction may be a risk factor for the development of PD-related peritonitis. Previously, there have been three case reports regarding acute peritonitis in patients not undergoing dialysis complicated with heart failure^44–46^, which supported our hypothesis.

Although our study included clinically stable patients (not presenting with overt volume overload status, or dyspnea due to heart failure), while performing UCG, patients with reduced LVEF may have demonstrated a constant hypervolemic status because the patients with reduced LVEF showed a higher cardiothoracic ratio on chest radiography, BNP, IVC, and lower IVCC, which are considered surrogate findings for fluid overload status^43^. Our results suggest that visceral congestion may be an important cause of enteric peritonitis.

Currently, there are no standardized criteria for the classification of organisms in peritonitis. In this study, enteric organisms were defined based on a previous report^33^ due to their predilection for intestinal colonization. However, we were unable to determine whether peritonitis caused by enteric organisms was certainly due to a bowel source and whether peritonitis caused by the non-enteric organisms was due to touch contamination or exit-site infection. Therefore, new testing techniques are required to confirm the origin of the causative organism^6,33,47^. In addition, in the present study, two patients developed peritonitis due to enteric organisms caused by ileus and diverticulitis, and the organisms responsible for it were *Escherichia coli* and *Proteus mirabilis,* respectively; these patients were included in the preserved LVEF group. Therefore, if these patients had been excluded from the statistical analysis, our results would remain unchanged. Furthermore, only three patients, one in the reduced LVEF group and two in the preserved LVEF group, had concomitant exit-site infection or tunnel infection-related peritonitis caused by *Staphylococcus aureus*. Based on these results, we propose that the bowel source may be important for the development of enteric peritonitis with cardiac dysfunction.

The present study had several limitations. First, given the retrospective nature of the study, unmeasured confounding factors associated with reduced LVEF may not have been included in the models. Second, this study had a single-center, small-cohort design; therefore, our results should be validated in studies with other large multicenter well-designed cohorts and longer follow-up periods. Third, heart failure is closely linked to poor dietary intake, malnutrition, low-level physical activity, and poor general condition^48^; therefore, the association with peritonitis may also be impacted by such conditions. As malnutrition is associated with immune defects, particularly a decrease in T cell function, it also contributes to an increased risk of and a worse outcome in cases of infections^49^. However, the present study could not evaluate these factors; consequently, the role of other confounding factors on the positive association between cardiac dysfunction and peritonitis cannot be ruled out and warrants further exploration. Fourth, this study could not detect changes in the intestinal microbacterial flora, and the pathomechanism of the development of peritonitis remains unknown. Fifth, the volume status and urine volume of each patient were not assessed during the follow-up. Furthermore, it is not known whether strict control of volume status could prevent the development of peritonitis. Further studies are therefore required to obtain this information.

Despite these methodological issues, to the best of our knowledge, this is the first study describing the relationship between LV function and peritonitis development. These results imply that patients with reduced LVEF may be at risk of enteric peritonitis from bowel sources caused by intestinal involvement due to cardiac dysfunction, which should be verified in different cohorts.

### Supplementary Information

Supplementary Figure S1.

Supplementary Table S1.

Supplementary Table S2.

## Data Availability

All relevant data are presented within the paper and its Supporting Information files.
