# Supplementary material for: Association between reduced left ventricular ejection fraction and peritoneal dialysis related peritonitis: a single center retrospective cohort study in Japan
Source: Sci Rep. 2023 Dec 20;13:22697. doi: 10.1038/s41598-023-49744-4 (PMC10733284; doi:10.1038/s41598-023-49744-4)
Supplement: Supplementary file 1 — Supplementary Figure S1. [file 41598_2023_49744_MOESM1_ESM.docx]

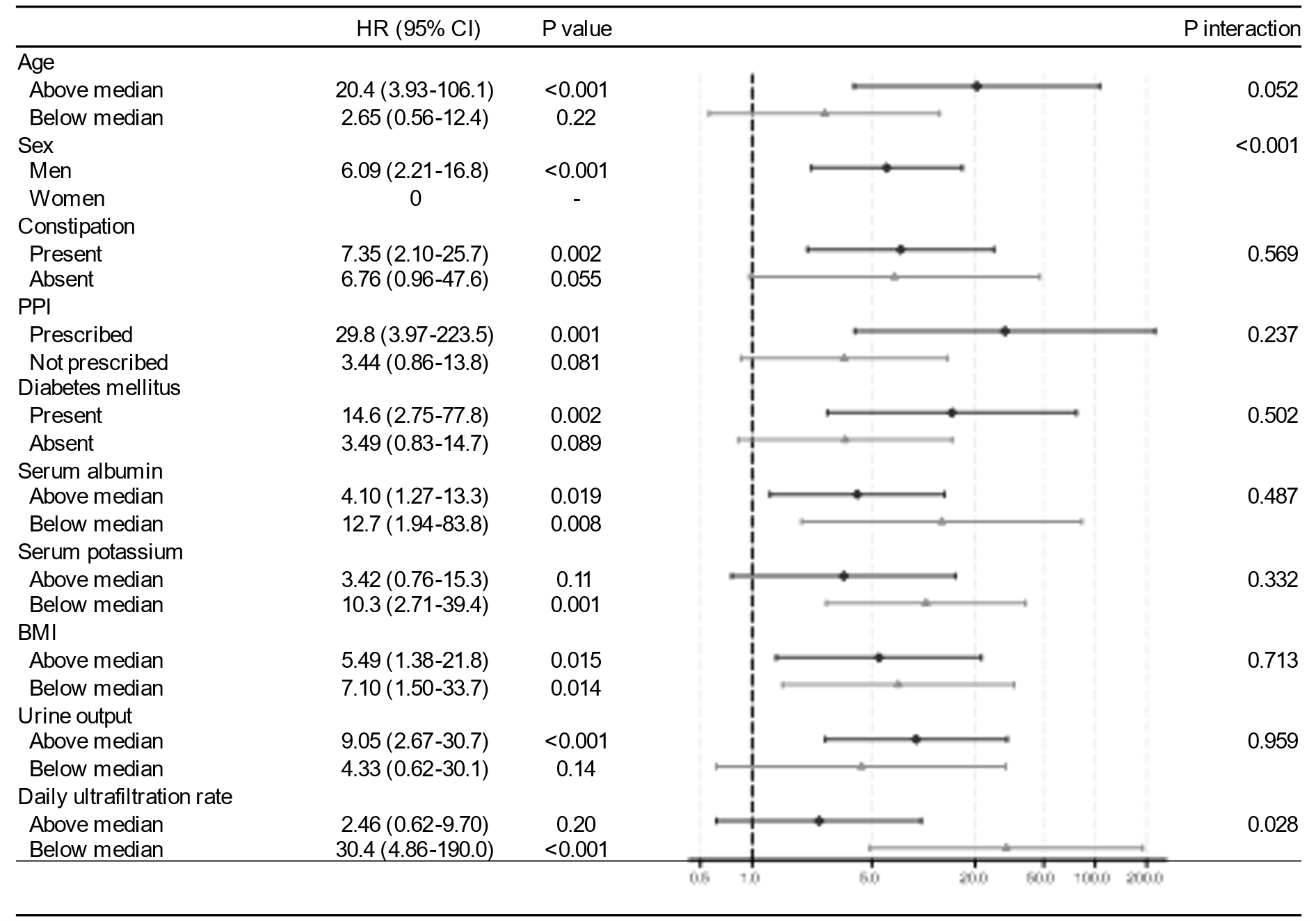


**Figure S1.** Stratified analysis of the association between reduced LVEF and enteric peritonitis

A forest plot demonstrating the HR for development of enteric peritonitis in each stratum, showing that similar associations throughout, except age, sex, and the daily ultrafiltration rate.
