# Supplementary material for: Association between reduced left ventricular ejection fraction and peritoneal dialysis related peritonitis: a single center retrospective cohort study in Japan
Source: Sci Rep. 2023 Dec 20;13:22697. doi: 10.1038/s41598-023-49744-4 (PMC10733284; doi:10.1038/s41598-023-49744-4)
Supplement: Supplementary file 2 — Supplementary Table S1. [file 41598_2023_49744_MOESM2_ESM.pdf]

TableS1. The anonymous data set of 228 patients with PD

No	Sex (ma	age	BMI	Alb	DM	K	Constipation	PPI	LVEF(%	all peritonitis (event:0)	Time to peritonitis (months)	enteric peritonitis	Time to enteric peritonitis (months)	non-enteric peritonitis	Time to non-enteric peritonitis (months)
1	2	35	20.5	4.9	0	4.4	0	0	55	1	105	1	105	1	105
2	1	26	23.6	4.4	0	3.2	0	0	69	1	87	1	87	1	87
3	2	41	22.2	3.9	0	3.1	0	0	38	0	21	1	129	0	21
4	2	43	21.9	3.9	0	5.2	0	0	65	0	48	1	97	0	48
5	1	35	21.2	3.8	1	4.7	0	0	43	1	97	1	97	1	97
6	2	35	19	4.1	0	3.8	1	0	59	1	96	1	96	1	96
7	1	31	26.6	4.6	0	4.1	1	0	59	0	1	1	38	0	1
8	1	49	20.3	3.7	0	4.4	1	0	73	1	56	1	56	1	56
9	2	46	28.9	3.4	1	3.4	0	1	68	1	77	1	77	1	77
10	1	59	20.5	3.6	1	3.5	1	1	38	0	21	0	21	1	77
11	1	59	23.1	3.9	1	3.5	1	0	64	0	50	1	76	0	50
12	2	38	34.2	4.2	1	3.6	1	0	80	1	47	1	47	1	47
13	1	65	23.3	3.5	1	3.7	1	0	70	0	7	1	70	0	7
14	2	54	19.5	3.5	1	3.3	1	1	62	0	5	1	32	0	5
15	1	62	21.4		1		1	0	41	0	3	0	3	1	23
16	1	49	23.4	4.1	0	4.9	1	1	35	1	57	1	57	1	57
17	1	49	23.5	3.4	1	6.8	1	0	43	0	13	1	55	0	13
18	1	67	21.5	3.4	0	5.4	1	0	76	1	34	1	34	1	34
19	1	74	20.6	3.2	1	3.6	1	0	49	1	51	1	51	1	51
20	1	48	29.7	3.9	0	3.9	0	0	61	0	48	0	48	1	87
21	1	76	19.2	3.7	0	5.1	1	0	44	0	51	1	70	0	51
22	1	76	22.1	3.3	0	3.5	1	1	62	1	48	1	48	1	48
23	1	63	23.6	3.4	0	3.6	1	0	62	1	47	1	47	1	47
24	2	72	20.7	3.6	0	4.9	1	0	76	1	87	1	87	1	87
25	1	61	20.1	2.6	0	3.6	0	0	54	1	46	1	46	1	46
26	1	72	19.9	2.4	0	4	1	0	58	0	4	1	55	0	4
27	1	59	22.7	3.4	1	4.5	0	0	44	1	29	1	29	1	29
28	1	81	18.1	2.1	1	3.1	1	1	50	0	0.7	1	24	0	0.7
29	2	65	22.8		0	4.7	0	0	42	1	57	1	57	1	57
30	1	57	24.1	3.8	1	5	1	0	61	1	25	1	25	1	25
31	1	64	31.3	3.2	1	3.6	1	0	40	0	2	1	12	0	2
32	1	60	28.2	3.8	1	3.9	1	1	55	0	1	1	32	0	1
33	2	55	27.6	3.4	0	4.2	1	0	49	1	90	1	90	1	90
34	2	66	17.5	2	1	3.7	1	1	72	1	19	1	19	1	19
35	1	75	23.5	2.3	1	3.5	1	0	37	1	1	1	1	1	1
36	1	59	24.7	3.9	1	4.2	1	0	73	1	44	1	44	1	44

37	1	67	20.9	3.7	1	4.2	0	0	63	1	31	1	31	1	31
38	2	58	18.8	4.2	1	4.3	1	0	65	0	40	1	71	0	40
39	2	80	23	3.3	1	4.1	1	1	63	0	52	1	61	0	52
40	2	58	19.9	4.8	0	3.6	1	1	58	1	59	1	59	1	59
41	2	69	22.2	3.1	1	4.4	1	0	56	0	10	1	31	0	10
42	1	64	23	2.9	0	4.5	1	0	57	0	32	1	38	0	32
43	1	73	20.6	3.8	1	4.9	0	0	46	1	37	1	37	1	37
44	1	71	22.4	2.7	1	3.5	1	1	48	0	2	0	2	1	3
45	1	61	20.8	4.3	0	4.1	1	0	52	1	77	1	77	1	77
46	2	80	19.3	2.8	0	3.5	1	0	78	0	33	1	41	0	33
47	1	67	20.6	2.3	1	4.5	1	1	61	0	50	1	68	0	50
48	1	53	22.6	3.2	1	5.9	1	0	54	1	26	1	26	1	26
49	1	70	23.3	3.8	1	4.4	1	0	69	1	56	1	56	1	56
50	2	72	15.1	3.2	1	4.6	1	0	76	0	24	1	46	0	24
51	1	64	17.5	3.5	0	3.7	1	0	62	1	44	1	44	1	44
52	1	48	22.6	3.8	1	3.2	1	1	72	1	45	1	45	1	45
53	1	61	25.2	3.9	0	5.5	1	1	52	0	46	0	46	1	49
54	2	33	17.3	3.6	0	3.3	1	0	58	0	17	1	65	0	17
55	2	56	25.6	4	1	4.3	1	0	64	1	58	1	58	1	58
56	1	37	24.5	4	0	5.3	1	0	62	0	3	0	3	1	17
57	1	63	19	3	0	2.8	1	0	60	0	23	0	23	1	24
58	1	56	28.5	3.9	1	3.2	1	0	69	0	5	1	63	0	5
59	1	47	30.5	2.9	1	6.1	1	1	36	0	15	1	65	0	15
60	1	53	20.6	4.1	1	5.8	1	0	49	1	21	1	21	1	21
61	1	64	22.2	4	0	5.2	1	0	35	1	56	1	56	1	56
62	1	37	22.1	4	0	4	1	0	61	1	11	1	11	1	11
63	2	33	20.9	3.5	1	4.6	1	0	78	1	75	1	75	1	75
64	1	64	22.8	3.3	1	3.5	1	1	56	1	27	1	27	1	27
65	1	37	19.3	3.9	0	4.9	1	1	42	0	65	0	65	1	68
66	1	73	21.6	2.7	1	3	1	0	83	0	16	1	28	0	16
67	1	63	18.2	4.1	0	4.2	1	0	64	1	76	1	76	1	76
68	1	47	23.2	3.7	1	5	1	1	55	0	20	1	36	0	20
69	2	73	21.6	2.3	0	2.6	1	1	58	0	4	1	22	0	4
70	1	61	21.2	3.6	1	4.5	1	0	61	0	21	1	64	0	21
71	1	74	21	2.9	1	3.5	1	0	52	1	44	1	44	1	44
72	1	63	24.3	2.5	1	3.5	1	0	65	1	64	1	64	1	64
73	2	57	20.3	3.7	1	3.9	1	0	57	0	37	0	37	1	47
74	1	75	20.6	2.9	1	3.2	1	0	55	1	38	1	38	1	38
75	1	60	34.2	3.1	1	4.8	1	0	79	1	51	1	51	1	51

76	2	77	27.6	3.4	1	3.2	1	1	74	0	24	1	19	0	24
77	1	58	19.8	3.9	1	3	1	0	57	1	93	1	93	1	93
78	1	67	26.4	4.1	1	4.6	1	0	68	0	27	0	27	1	29
79	1	61	23.6	3.9	0	3.1	1	0	60	1	76	1	76	1	76
80	1	59	24.3	2.6	1	4.3	1	0	47	1	47	1	47	1	47
81	2	50	20	3.1	1	3.4	1	0	80	0	10	1	53	0	10
82	1	56	25	3.2	0	4.6	1	0	58	1	53	1	53	1	53
83	1	79	19.9	3.7	0	3.8	1	0	56	1	40	1	40	1	40
84	2	63	17.3	2.8	1	4.3	0	1	56	1	48	1	48	1	48
85	1	76	21.6	3.7	1	4.4	1	0	55	0	5	1	7	0	5
86	1	62	18.7	4.1	0	6	1	0	75	0	4	1	6	0	4
87	1	67	23.7	4.3	0	4.1	1	1	69	0	57	1	57	0	57
88	1	44	39.5	3.8	0	5.2	1	0	77	1	19	1	19	1	19
89	1	59	23.9	3.2	0	3.1	1	1	56	1	33	1	33	1	33
90	1	67	23.2	3.1	1	5.2	0	1	61	0	24	1	32	0	24
91	2	62	27.9	2.9	1	2.8	0	1	57	0	29	1	54	0	29
92	1	67	30.1	3.4	1	4.8	1	1	73	0	2	1	34	0	2
93	1	45	21.1	2.5	1	4	1	1	37	0	0.03	1	13	0	0.03
94	1	65	15.6	2.7	1	4.3	0	0	55	1	1	1	1	1	1
95	2	68	16.3	3.9	0	4.1	0	0	56	1	29	1	29	1	29
96	1	68	24.2	4.4	1	6.6	1	0	80	1	23	1	23	1	23
97	2	64	26	4.2	0	4.2	1	0	53	1	25	1	25	1	25
98	2	42	23.9	4.2	0	4.1	1	0	52	1	44	1	44	1	44
99	1	65	29.5	4.1	0	4.3	0	0	82	1	41	1	41	1	41
100	1	80	20.1	2.6	1	3.4	1	1	73	0	4	1	33	0	4
101	1	83	21.8	3.9	0	4.8	1	0	47	1	10	1	10	1	10
102	2	73	19.2	3.1	0	4.7	1	0	58	1	8	1	8	1	8
103	1	43	23.4	2.9	1	4.1	1	1	42	0	25	1	53	0	25
104	2	75	22.3	3.1	0	4.5	1	0	78	1	33	1	33	1	33
105	1	75	22.8	2.6	0	3.2	1	0	65	1	16	1	16	1	16
106	1	37	17.9	4.1	0	5.4	1	1	74	1	21	1	21	1	21
107	1	39	27.9	3.6	1	3.9	0	0	57	1	35	1	35	1	35
108	1	64	24.2	3.8	1	3.9	0	1	80	1	30	1	30	1	30
109	1	54	19.9	4.1	0	4.4	1	0	66	1	42	1	42	1	42
110	1	56	27.9	3.7	1	4.9	1	0	62	1	26	1	26	1	26
111	1	72	27.4	3	1	5.4	0	0	38	0	0.9	0	0.9	1	18
112	2	36	17.3	3	0	5	0	0	68	1	20	1	20	1	20
113	1	46	28.3	2.8	1	4.6	0	0	66	1	15	1	15	1	15
114	1	64	19.5	3	1	4	0	0	56	1	48	1	48	1	48

115	1	39	27	3.4	0	6.4	0	1	72	0	9	1	27	0	9
116	1	53	31.5	3.9	0	4.1	1	1	74	1	32	1	32	1	32
117	1	84	24.5	2.1	1	3.7	0	0	72	1	15	1	15	1	15
118	1	28	23	4.1	0	3.8	1	0	55	1	20	1	20	1	20
119	1	60	30.4	2.2	1	3.5	1	0	76	1	14	1	14	1	14
120	1	62	24	3.9	1	3.8	0	0	51	1	39	1	39	1	39
121	1	78	19.1	2.7	1	4.1	1	0	74	1	21	1	21	1	21
122	1	44	23.3	2.2	1	3.3	0	1	46	0	0.27	1	20	0	0.27
123	2	61	21.5	3.5	0	4.8	0	0	38	1	7	1	7	1	7
124	2	24	30.7	3.2	1	5.5	1	0	58	0	1	1	6	0	1
125	2	64	28.4	3.1	1	4.7	1	0	59	1	2	1	2	1	2
126	1	40	21.5	4	0	5.4	0	0	64	0	21	1	11	0	21
127	2	55	20.2	2.6	0	5.1	1	0	62	1	18	1	18	1	18
128	1	58	22.7	2.9	0	3.9	0	1	72	0	4	1	12	0	4
129	1	67	27.2	2.8	0	3.7	1	1	73	0	16	1	25	0	16
130	1	64	20.8	1.9	1	3.9	1	1	80	1	3	1	3	1	3
131	1	55	32.2	3.1	1	5.2	0	0	60	1	14	1	14	1	14
132	1	76	23	4.1	0	4.3	0	1	39	0	13	0	13	1	11
133	1	62	22.2	2.7	0	4.1	1	1	72	1	16	1	16	1	16
134	2	56	21.8	1.5	0	6	1	0	76	1	10	1	10	1	10
135	1	79	26.7	3	1	3.5	1	0	75	0	7	1	6	0	7
136	1	75	25.4	3.5	1	3.2	0	0	67	1	7	1	7	1	7
137	1	75	19.6	2.4	1	4.3	0	0	69	0	1	1	17	0	1
138	1	81	24.8	2.7	1	3.6	0	1	55	0	12	1	17	0	12
139	1	67	20.5	3.1	0	4.7	1	1	64	1	13	1	13	1	13
140	1	66	19.7	2.5	0	5.6	1	0	71	1	0.5	1	0	1	0
141	1	67	22.3	4	1	5.3	0	0	67	1	60	1	60	1	60
142	1	70	18.1	3.5	0	4.8	0	0	75	0	4	1	14	0	4
143	2	51	37.1	3.2	1	2.7	1	0	71	1	31	1	31	1	31
144	1	44	25.9	3.2	1	4.7	0	0	75	1	5	1	5	1	5
145	1	75	18.2	2.5	0	2.9	1	0	76	1	8	1	8	1	8
146	2	67	20.7	3.7	1	4.1	0	1	57	0	6	1	53	0	6
147	1	38	27.2	2.7	1	4.4	0	0	76	1	19	1	19	1	19
148	1	52	19.5	3.4	1	4.4	1	1	52	1	73	1	73	1	73
149	1	76	19.2	3.7	0	4.3	0	0	67	1	60	1	60	1	60
150	1	61	34.3	4.1	1	3.4	1	1	51	1	16	1	16	1	16
151	2	47	19.3	4	0	4.4	1	1	65	1	78	1	78	1	78
152	1	60	20.8	3.8	1	4.1	0	1	62	0	100	0	45	1	52
153	1	73	17.2	2.8	0	5.2	1	1	51	0	45	1	71	0	1

154	1	70	18.6	3.4	1	4.3	1	1	70	1	52	1	52	1	52
155	1	70	22	3.8	0	3.2	1	0	68	1	24	1	24	1	24
156	1	54	24.7	4.2	0	4.1	1	0	77	0	16	1	60	0	16
157	1	61	29.6	3	1	3.7	1	0	74	1	24	1	24	1	24
158	1	72	19.8	3.7	0	4.9	0	1	83	1	55	1	55	1	55
159	1	75	24.5	3.1	0	3.4	1	0	76	1	16	1	16	1	16
160	2	49	25.3	3.8	0	4.4	1	0	65	1	40	1	40	1	40
161	1	89	22.1	3.6	1	4.5	0	1	80	0	1	1	21	0	1
162	2	64	19.3	3.4	0	3.5	1	0	69	1	90	1	90	1	90
163	1	72	21.3	3.5	0	5.7	1	1	68	0	4	1	33	0	4
164	1	72	20.2	3.3	1	4.3	1	1	80	0	41	1	46	0	41
165	1	81	23.8	3.4	0	4	1	0	77	1	24	1	24	1	24
166	1	56	23.3	4.1	0	5.5	1	0	66	1	29	1	29	1	29
167	1	48	24	3.9	0	4.5	1	1	80	1	19	1	19	1	19
168	1	63	23.3	3.7	0	3.8	1	0	88	0	70	1	92	0	70
169	2	76	25.5	2.8	0	4.7	1	0	66	1	17	1	17	1	17
170	2	67	21.2	2.8	0	2.4	1	1	51	0	26	1	44	0	26
171	1	64	18.9	3.4	0	4.5	1	0	64	1	27	1	27	1	27
172	2	75	20.5	3.7	0	4.8	0	1	57	0	39	1	72	0	39
173	1	69	19.1	3.5	1	3.1	1	1	68	0	28	1	60	0	28
174	2	60	22.6	3.4	1	5.8	1	0	69	1	59	1	59	1	59
175	1	74	26.7	4	1	6.1	1	0	67	1	20	1	20	1	20
176	2	64	23.6	3.8	0	3.2	1	0	61	1	121	1	121	1	121
177	1	60	20.6	2.5	1	3.6	0	0	66	1	46	1	46	1	46
178	1	77	19.5	3.5	0	3.2	1	0	77	1	42	1	42	1	42
179	1	63	25.3	3.7	0	4.2	1	0	73	1	44	1	44	1	44
180	1	60	24.1	3.6	1	3.5	1	1	73	0	9	1	59	0	9
181	1	68	24.7	3.2	1	4.3	0	0	68	1	10	1	10	1	10
182	1	75	18.6	3.6	0	4.6	1	0	69	1	30	1	30	1	30
183	1	74	25.1	3.8	0	4.4	0	0	65	1	58	1	58	1	58
184	1	57	20.4	3.4	0	4.4	1	0	69	1	34	1	34	1	34
185	2	74	19	3.4	0	3.2	1	1	78	1	53	1	53	1	53
186	1	62	31.1	3.3	1	5.9	0	0	63	1	45	1	45	1	45
187	1	68	21.5	3.7	0	4.3	1	0	54	0	35	1	91	0	35
188	1	71	23.6	4.1	0	3.7	1	0	20	0	0.97	0	0.97	1	17
189	1	80	24	3.3	0	5	1	0	71	1	33	1	33	1	33
190	2	61	23.9	2.6	1	4.6	0	0	75	0	8	1	14	0	8
191	1	68	21.3	2.4	1	3.6	1	1	49	0	27	0	27	1	32
192	1	81	19.1	2.6	0	5.8	1	0	51	1	25	1	25	1	25

193	1	53	25.1	3.4	0	3.7	0	1	75	1	17	1	17	1	17
194	1	77	21.1	2.8	1	3.7	1	1	73	1	61	1	61	1	61
195	1	65	17	3.3	0	4	0	0	58	0	27	0	27	1	64
196	1	63	23.4	3	0	3.9	0	1	65	1	66	1	66	1	66
197	2	73	20.5	3.1	0	4	0	0	71	1	40	1	40	1	40
198	1	58	22.9	3.6	0	4.9	1	0	78	1	31	1	31	1	31
199	1	66	15.8	3	1	4.7	1	0	73	0	3	1	55	0	3
200	2	66	32	3.2	1	3.5	0	0	77	1	22	1	22	1	22
201	2	66	23.7	3.8	1	4.3	0	1	65	1	99	1	99	1	99
202	1	62	21	3.8	1	4.2	1	0	55	0	25	1	52	0	25
203	2	72	33.2	3.8	1	4.2	1	1	70	0	44	1	58	0	44
204	2	45	16.2	4.2	0	5.5	0	1	78	1	57	1	57	1	57
205	1	71	22.4	3.5	1	3.8	1	0	65	1	43	1	43	1	43
206	2	74	22.1	4.2	0	3.5	1	0	78	1	28	1	28	1	28
207	1	84	16.9	3.8	0	5.6	1	1	64	1	39	1	39	1	39
208	1	66	19.4	3.2	1	3.1	1	0	76	1	21	1	21	1	21
209	1	42	20.6	4.5	0	3.4	1	0	70	1	49	1	49	1	49
210	1	71	21	3.8	1	4.2	0	0	58	0	15	1	25	0	15
211	1	58	25.5	3.5	0	4.9	0	1	65	0	54	0	54	1	74
212	1	66	24.1	2.9	0	4	1	0	73	1	36	1	36	1	36
213	2	60	28.5	3.9	1	4.1	1	1	65	0	69	1	68	0	69
214	1	44	21.3	4.2	1	5.1	0	1	62	1	63	1	63	1	63
215	1	74	22.2	3.4	1	4.8	1	1	71	1	14	1	14	1	14
216	2	49	16	3.2	0	3.9	1	0	77	1	19	1	19	1	19
217	2	81	23.3	3.5	0	4.2	1	0	59	1	33	1	33	1	33
218	1	63	33.2	4	0	3.4	1	0	58	1	14	1	14	1	14
219	1	71	21.4	3.1	1	4.5	1	0	70	1	14	1	14	1	14
220	2	64	18.3	3	0	3.5	1	1	71	1	63	1	63	1	63
221	2	67	17.2	4	0	4.4	0	0	70	1	47	1	47	1	47
222	1	82	18.7	2.7	0	5.2	1	1	56	0	22	1	26	0	22
223	1	79	20.5	2.6	0	4.3	1	0	66	1	7	1	7	1	7
224	1	84	18.9	3.1	0	4.7	1	0	63	1	3	1	3	1	3
225	2	79	26.9	3	0	3.1	1	0	75	1	12	1	12	1	12
226	2	80	18.7	2.4	0	3.6	0	0	63	1	5	1	5	1	5
227	2	83	17.5	3.2	1	2.6	0	1	73	1	11	1	11	1	11
228	2	87	18.6	2	0	2.9	1	0	48	0	2	1	12	0	2
