# Supplementary material for: Association between reduced left ventricular ejection fraction and peritoneal dialysis related peritonitis: a single center retrospective cohort study in Japan
Source: Sci Rep. 2023 Dec 20;13:22697. doi: 10.1038/s41598-023-49744-4 (PMC10733284; doi:10.1038/s41598-023-49744-4)
Supplement: Supplementary file 3 — Supplementary Table S2. [file 41598_2023_49744_MOESM3_ESM.docx]

**Table S2. Comparison of causes of PD withdrawal**

|  | Reduced LVEF group (LVEF<50%)  (n = 30) | Preserved LVEF group (LVEF≥50%)  (n = 198) |
| --- | --- | --- |
| **PD withdrawal** | 22 (73.3) | 145 (73.3) |
| PD-related infection | 2 (9.1) | 25 (17.2) |
| Inadequate solute clearance | 4 (18.2) | 50 (34.5) |
| ADL impairment | 1 (4.5) | 14 (9.7) |
| Renal transplantation | 3 (13.6) | 2 (1.4) |
| Fluid overload | 5 (22.7) | 18 (12.4) |
| Death | 6 (20.0) | 23 (11.6) |
| Cardiovascular disease | 5 | 8 |
| Malignancy | 1 | 1 |
| Infection | 0 | 8 |
| Others | 0 | 6 |
| Others | 1 (4.5) | 13 (9.0) |

Categorical values are expressed as numbers (proportions).

Abbreviations: PD, peritoneal dialysis; ADL, activities of daily living
